# Supplementary material for: Exploring the key points and key shots in table tennis matches based on survival analysis
Source: PLoS One. 2026 Apr 22;21(4):e0338203. doi: 10.1371/journal.pone.0338203 (PMC13102243; doi:10.1371/journal.pone.0338203)
Supplement: Attachment — Submitted filename: Response to Reviewers.docx [file pone.0338203.s002.docx]

Reviewer #1:

We sincerely thank Reviewer #1 for their valuable time and constructive feedback. These comments have greatly helped us improve the quality of our manuscript. We have carefully revised the manuscript in response to each of your comments. Our specific responses are as follows:

Abstract:

-The statistical method used is not stated in the abstract or method.

Reply: We have now explicitly stated the statistical methods used—namely, the “Kaplan-Meier method” and the “Log-rank (Mantel-Cox) non-parametric test” in the abstract. Additionally, a new subsection titled “Statistical Analysis” has been added to the Methods chapter to provide a detailed description of the statistical approaches.

Method:

-How were the selected matches selected?

Reply: All match samples were selected from: World Championships, World Cups, Olympic Games, and WTT Champions tournaments. Based on the ITTF world rankings, matches were further categorized into Balanced, Unbalanced, and Blowout groups (see “Classification of groups” for details) to examine variations in *KP* and *KS* across different competitive levels.

-What criteria did you use while watching? For example, was it considered whether the athlete was right-handed or left-handed?

Reply: Data were collected by two trained players from the Chinese Table Tennis Association (CTTA), with an emphasis on core indicators such as match outcome, points won/lost and shot counts. The study included both right-handed and left-handed players to ensure comprehensive coverage.

-The statistical method used is not stated in the abstract or method.

Reply: Firstly, this revision places greater emphasis on the statistical analysis methods employed in this study within the abstract section. Secondly, in the “Methods” chapter, we have restructured the organization: the “Statistical Analysis” subsection has been added. This enables readers to gain a clearer understanding of the statistical techniques utilized throughout the study.

-Which age group was the study conducted for? And where was it conducted?

Reply: The study focused on elite table tennis players, with data sourced from three premier international tournaments that do not organize competitions by age group. Female athletes in this study ranged in age from 20 to 38 years old last year, while male athletes ranged from 21 to 48 years old.

-How were the observation forms created? Was expert support obtained?

Reply: The performance analysis framework was adapted from the methodology proposed by Prof. Tamaki S, which incorporates shot count as a key metric. This approach has been validated in peer-reviewed literature (Ref. [10] DOI: 10.1515/hukin-2017-0002) and is recognized in the field.

-How was cumulative survival probability calculated? Was it calculated by an expert?

Reply: The cumulative survival probability was calculated using the Kaplan-Meier method, a standard tool in survival analysis. All calculations were performed with SPSS 27.0 and GraphPad Prism 9.0, as detailed in the “Methods” section.

-There are formulas in the method section, whose formulas are these? No source.

Reply: We have now provided authoritative references for all key formulas used in the Methods section.

-What is the score of the Log-Rank nonparametric test? Why is it nonparametric?

Reply: The Log-rank test is a non-parametric method suitable for comparing survival curves without assuming a specific distribution. The p-values were derived by comparing observed and expected event counts, as detailed in the “Methods” section.

Findings

The findings include 0.05 and 0.01. How was this calculated? It should be stated.

Reply: The p-values (0.05, 0.01) were obtained from the Log-rank test, indicating the significance of differences between survival curves. The calculation is explained in the “Methods” subsection.

Discussion

-Why were psychological states included in the discussion without adhering to a specific criterion? The psychological states of the athletes were mentioned in the discussion. How were the athletes' psychological states determined? Was a scale used?

Reply: The discussion of psychological states was based on theoretical and literature support, not direct measurement. We have acknowledged this limitation in the revised Discussion and Limitations sections.

Suggestions

-The suggestions are insufficient; they should be rewritten.

Reply: We have thoroughly revised the “Suggestions” section to provide more actionable and context-specific recommendations for training, match strategy, and potential rule changes.

Reviewer #2:

We sincerely thank Reviewer #2 for affirming our work and providing insightful and constructive feedback. Your high praise has greatly encouraged us, and your detailed revision suggestions have significantly enhanced the clarity and scientific rigor of our manuscript. We have revised the manuscript in response to each of your comments. Our specific responses are as follows:

Abstract

L33: Please clarify which matches were analyzed (e.g., tournament, championship, competition level).

Reply: We have specified that all matches were drawn from the World Championships, World Cup, and Olympic Games, representing the highest level of international table tennis.

L34: Readers may not be familiar with survival analysis. Please provide a brief definition or explanation, while considering the word limit.

Reply: A concise explanation of survival analysis and its application to table tennis has been added to the Abstract and Methods sections.

L42–45: Refine the conclusion so that it more directly reflects the current findings.

Reply: We have revised the results and conclusions section of the abstract to present the core research findings more directly, thereby emphasising the theoretical and practical significance of this study’ s outcomes.

Introduction

L72: Please double-check for a possible typo.

Reply: This revision has undergone thorough proofreading, identifying and correcting all spelling errors to ensure the text flows smoothly.

L72: Specify which researchers are being referred to.

Reply: This revision clarifies the researchers cited and their respective contributions.

L78: The discussion shifts to badminton. After presenting other sports, the focus should return to table tennis.

Reply: This revision has incorporated relevant discussion points to ensure timely return to the main thread of table tennis research following references to other projects.

L82: Provide a reference to support this claim.

Reply: Relevant literature has been supplemented to support the claims made in the text. Reference: Wang C-C. A Binary-Entropy Analysis of the Relationship Between Scoring Structure and Match Outcome in Badminton. Frontiers in Psychology. 2022;13. DOI: 10.3389/fpsyg.2022.799293.

L83: Define which “comparative studies” are mentioned.

Reply: This revision has incorporated relevant literature citing “comparative research” within the text, clarifying that “comparative research” denotes comparative analysis of technical and tactical performance between Chinese and foreign athletes. Additional references:

(1) Kong L, Ding N, Wu F. Preparation for 2024 Paris Olympic Games: Analysis of the Technical and Tactical Characteristics of the World's Elite Players by UsingABS Table Tennis Ball. Journal of Sports and Science. DOI:10.13598/j.issn1004-4590.2023.02.012.

(2) Xu D. Technical and Tactical Characteristics of Fan Zhendong and TrulsMoregard in the Table Tennis Men’s Singles Final at the Paris Olympics A Comparative Analysis based on the Four - Phase Index Evaluation Method. Bmc Sports Science Medicine and Rehabilitation. 2025;33(01):4–6+31. DOI:10.19379/j.cnki.issn.1005-0256.2025.01.002.

(3) Lanzoni I, Di Michele R, Merni F. A notational analysis of shot characteristics in top-level table tennis players. European Journal of Sport Science. 2014;14(4):309–17. DOI:10.1080/17461391.2013.819382

(4) Qing Yang, Mu-zi Li, Zheng Zhou and Hui Zhang*. Exploring the structure of the shot effectiveness model for elite table tennis players [J]. BMC Sports Science, Medicine and Rehabilitation, 2023, 15:127. <https://doi.org/10.1186/s13102-023-00736-x.>

L104: Clearly state the study hypotheses.

Reply: The research hypotheses have been proposed at the conclusion of the introduction in this revision.

I recommend incorporating the following references to strengthen the introduction and discussion:

Reply: We are grateful to the Professor for the reference list provided. The aforementioned literature has been incorporated into this revision to strengthen the arguments presented in the introduction and discussion sections.

Materials and Methods

L106: Provide more detail about which matches were analyzed (e.g., type, level, and selection criteria).

Reply: This revision has added detailed information regarding competition selection to the “Match samples” subsection, including event types, levels, and grouping criteria.

L127: Please re-check this line for accuracy.

Reply: This revision has reexamined the accuracy of this line and corrected the existing errors. It should be noted that in table tennis terminology, a “Game” refers to a single set within a match (typically played to 11 points), while the overall contest is referred to as a “Match”. In this study, we analyzed games within matches to identify *KP*.

L143: Consider restructuring the methods section by introducing a “Statistical Analyses” subsection first, and then describing survival analysis and other methods.

Reply: This revision has restructured the document as recommended, moving the ‘Statistical Analysis’ section to the front.

Results

The results and tables are generally well-presented. Congratulations on this clear presentation.

Reply: We are grateful for the Professor’s affirmation of the “Results” section, which has greatly encouraged our subsequent research endeavours.

Discussion

The discussion is well-structured, but several points require attention:

L286: Begin the discussion with a short paragraph summarizing the main results before introducing sub-sections.

Reply: This revision has incorporated the recommendation by adding an introductory paragraph to the “Discussion” section that concisely summarises the principal findings. It clearly outlines the core discoveries regarding *KP* and *KS*, followed by detailed discussions in two sub-sections: “Key points and their significance in table tennis” and “Key shots and their significance in table tennis”.

The emphasis on the psychological role of key points (KP) is strong, but there is no empirical evidence on how emotions or tactics actually shift at KP. Please acknowledge this gap.

Reply: This revision has been explicitly stated in the Discussion and Limitations section.

The connection between KP thresholds and psychological/tactical execution remains hypothetical; this should be noted as a limitation.

Reply: This revision has been explicitly stated in the limitations section.

Women’s matches are observed to have longer key shots (KS) in close games, but the reasons (technical, tactical, psychological) are not sufficiently explored.

Reply: We sincerely thank the Professor for this insightful comment. As rightly pointed out, our initial manuscript did not sufficiently explore the reasons behind the prolonged KS in women’s matches during decisive games, in contrast to the absence of such a trend in men’s matches.

In response, this revision includes a newly added paragraph in the corresponding section of the “Key shots and their significance in table tennis” chapter. From the theoretical perspective of “risk-coping strategies,” this addition delves into the tactical and psychological underpinnings of this gender-based disparity. We propose that female athletes tend to adopt a risk-aversion strategy under high-pressure situations, managing risk by prolonging rallies and minimizing unforced errors. In contrast, male athletes lean toward a risk-taking approach, relying more on shot quality to actively score points. This supplementary content provides a robust explanation for the observed phenomenon and enriches the theoretical depth of the paper.

The limitations section could be expanded to acknowledge:

Player-specific factors (e.g., style, handedness, ranking, tactical preferences). Lack of psychological or physiological data (e.g., emotional state, heart rate, perceived pressure). Simplifications of match dynamics inherent in survival analysis.

Reply: This revision has supplemented the “Limitations” section, clarifying the study’s constraints regarding individual athlete variations, the inherent simplification of competition dynamics in survival analysis, and psychological and physiological data.

Finally, we extend our sincere appreciation to the editors and reviewers for their thorough and constructive feedback, which has been instrumental in refining both the research and its presentation. In response to each of the suggestions raised, the authors have carefully revisited the manuscript and made extensive revisions to strengthen the depth of analysis, logical coherence, and clarity of expression. While every effort has been made to address the points raised, we recognize that there may still be aspects that can be further improved. We warmly welcome any additional comments and kindly request a re-evaluation of the revised manuscript. We apologize for any additional workload this process may have incurred and once again express our deepest gratitude. We look forward to the continued guidance of the editors and reviewers in helping this study reach its full potential.

Sincerely,

October 29, 2025
